# Advancing Sustainability through Urban Green Space: Cultural Ecosystem Services, Equity, and Social Determinants of Health

**DOI:** 10.3390/ijerph13020196

**Published:** 2016-02-05

**Authors:** Viniece Jennings, Lincoln Larson, Jessica Yun

**Affiliations:** 1 Southern Research Station, Integrating Human and Natural Systems, USDA Forest Service, 320 Green Street, Athens, GA 30602, USA; 2 Department of Parks, Recreation, and Tourism Management, Clemson University, Clemson, SC 29634, USA; lrl@clemson.edu; 3 Department of Science, Technology and International Affairs, Georgetown University, Washington, DC 20057, USA; jjy24@georgetown.edu

**Keywords:** ecosystem services, green space, nature, parks, public health, urban health

## Abstract

Urban green spaces provide an array of benefits, or ecosystem services, that support our physical, psychological, and social health. In many cases, however, these benefits are not equitably distributed across diverse urban populations. In this paper, we explore relationships between cultural ecosystem services provided by urban green space and the social determinants of health outlined in the United States Healthy People 2020 initiative. Specifically, we: (1) explore connections between cultural ecosystem services and social determinants of health; (2) examine cultural ecosystem services as nature-based health amenities to promote social equity; and (3) recommend areas for future research examining links between urban green space and public health within the context of environmental justice.

## 1. Introduction

The World Health Organization defines health as “a state of complete physical, mental and social well-being, and not merely the absence of disease or infirmity” [1]. Healthy People 2020, an initiative from the U.S. Department of Health and Human Services, also embraces this holistic view of health and well-being by highlighting the interaction between factors (e.g., biological, social, and behavioral) that collectively influence individual and community health [2]. Compared to previous versions of this initiative, Healthy People 2020 extends the dialogue by explicitly acknowledging and emphasizing social determinants of health (e.g., community context, the built environment, and socio-economic standing) as key factors in health promotion [3]. A focus on underlying social aspects can help the public health community strategically address multiple health objectives in a socially equitable way [3,4,5,6]. Since social equity represents a recurring theme in the literature on social determinants of health [4,5,6] and more scholars note how these factors contribute to health disparities [7,8], these trends highlight important yet overlooked connections that warrant further exploration. We aim to bridge this knowledge gap by discussing the connections between social determinants of health, social equity, and the cultural ecosystem services derived from green space in urban areas.

Social equity can be defined as “the fair, just and equitable management of all institutions serving the public directly… and the commitment to promote fairness, justice, and equity in the formation of public policy.” [9]. This concept also fueled the environmental justice movement, which was historically focused on health consequences associated with inequitable distribution of environmental hazards in low-income and/or racially/ethnically diverse communities [10,11]. However, recent literature has expanded traditional thinking on environmental justice by emphasizing positive contributions of natural environments to health and well-being [12,13]. It has also connected the inequitable distribution of these nature-related benefits to health disparities frequently observed across socio-demographic boundaries [14]. Despite this shifting perspective, inequitable access to and enjoyment of these nature-related benefits remains a prominent barrier to sustainable development [14,15]. Reexamining these benefits in the context of human health and well-being may represent an important step in the environmental justice movement.

The natural environment, often described as “green space” in urban areas (e.g., forests, parks, gardens, greenways), is widely considered to be an important contributor to health [16,17,18,19,20]. Green spaces provide indirect and direct benefits to human health and well-being, which are often described as ecosystem services [21,22,23]. These services are classified in a variety of ways. While some scholars use the terms ecosystem services and benefits synonymously [24], others argue that outputs should not be classified as “services” unless they generate benefits valued by society [25,26]. As we acknowledge both perspectives, we chose the broader definition of ecosystem services, outlined in frameworks such as the Millennium Ecosystem Assessment and Common International Classification of Ecosystem Services, which incorporates both intermediate and final goods and services. The most recent framework [23] uses the categories of provisioning services (e.g., food sources, biomass), regulating and maintenance services (e.g., decomposition, water filtration, climate regulation), and cultural services (e.g., aesthetic value, outdoor recreation).

A substantial body of work has also examined the contributions of provisioning, regulatory, and maintenance ecosystem services to urban health. For example, urban green space can regulate air and water pollution [27], mitigate urban heat effects [28], and enhance access to nutritious fruits and vegetables [29,30], all of which enhance the physical health of urban residents. Meanwhile, the influence of cultural ecosystem services has been more difficult to assess, in part because these services generate “non-material” benefits that produce less tangible impacts [31,32]. Research indicates that the benefits from cultural services (e.g., landscape aesthetics, outdoor recreation, spiritual and cultural values) are no less important to health and well-being, yet their value may be frequently underestimated [31,33] For example, outdoor recreation can increase a population’s level of physical activity and potentially reduce the risk of chronic health conditions such as obesity or cardiovascular disease [34,35,36]. Aesthetic immersion in natural landscapes can also reduce stress and anxiety in addition to enhance human capacity for physical and spiritual restoration [37,38,39,40]. Theoretically, ecosystem services have the potential to generate similar benefits across all segments of the human population. However, because ecosystem services (in general) and cultural services (in particular) are not evenly distributed across urban landscapes, differential access to and use of green space can exacerbate health disparities [14,41,42]. Enhanced understanding of cultural ecosystem services and the benefits they generate across diverse urban landscapes could therefore help to inform health-related policy and decision making.

In this paper, we examine an emerging frontier in environmental justice: the movement to ensure that urban ecosystem services and the health benefits they provide are equitably distributed among all segments of the population. Such an approach can inform how we assess environmental conditions within communities and achieve mutual goals of environmental and public health professionals [43]. We begin by synthesizing recent literature to illustrate important links between urban green space, cultural ecosystem services, and social determinants of health (Table 1). According to Healthy People 2020, these social determinants include: (1) health and health care; (2) neighborhood and built environment; (3) social and community context; (4) education; and (5) economic stability [2]. We then discuss this topic in the context of environmental justice, focusing on the distributional equity of urban green space and corresponding ecosystem services across disadvantaged communities. Finally, we highlight opportunities for future research and policy initiatives to link benefits from green spaces with equity and social determinants of health.

**Table 1:** Connections between social determinants of health and benefits linked to cultural ecosystem services provided by urban green spaces.

Social Determinant of Health	Benefits linked to Cultural Ecosystem Services	Example Studies on Urban Green Space and Domains of Health/Well-Being
Health and health care	Physical Well-Being	West *et al.*, 2012 [44], Cohen *et al.*, 2007 [45], Kaczynski *et al.*, 2014 [46]; Floyd *et al.*, 2011 [47]
	Psychological Well-Being	Cohen-Cline *et al.*, 2015 [48]; Beyer *et al.*, 2014 [49]; Berman *et al.*, 2012 [50]
Neighborhood and built environment	Sense of Place	Harper *et al.*, 2012 [51]; Peters *et al.*, 2010 [52]
	Community Satisfaction	Hur *et al.*, 2010 [53]; Vemuri *et al.*, 2009 [54]
	Reduced Crime and Incivilities	Kondo *et al.*, 2015 [55]; Branas *et al.*, 2011 [56]; Kuo *et al.* 2001 [57]; Bogar & Beyer 2015 [58]
	Access to Healthy Food	Comstock *et al.* [59]; Litt *et al.* [29]; McLain *et al.*, 2014 [60]
Social and community context	Social Cohesion	Fan *et al.*, 2011 [61]; Sugiyama *et al.*, 2008 [62]
	Social Capital	Holtan *et al.*, 2014 [63]; Zelenski *et al.*, 2015 [64]
Education	Academic Performance	Schutte *et al.*, 2015 [65]; Wu *et al.*, 2014 [66]; Matsuoka, 2010 [67]
	Cognitive Functioning	Dadvand *et al.*, 2015 [68]; Kuo *et al.*, 2004 [69]
Economic Stability	Property Values	Conway *et al.*, 2010 [70]; Voicu and Been, 2008 [71]; Kovacs, 2012 [72]
	Community Revitalization	Branas *et al.*, 2011 [56]; Schilling and Logan, 2008 [73]
	Socioeconomic Status	Schwarz *et al.*, 2015 [74]; Bruton and Floyd, 2014 [13]; Duncan *et al.*, 2013 [75]; Landry and Chakraborty, 2009 [76]

## 2. Connections between Social Determinants of Health and Urban Green Space

### 2.1. Health and Health Care

The category of health and health care pertains to a social determinant that involves health literacy, access to health care, and access to primary care [2]. Though the concept generally refers to conventional medical treatment and physician care, proactive upstream health interventions have become increasingly important within this sector. For instance, an approach oriented to ecosystem services may be a promising way to translate the benefits from green spaces into preventive health strategies [77]. This line of thinking is reflected in emerging initiatives such as Park Prescription programs (Park or Nature Rx), where medical professionals prescribe “time outdoors” to enhance physical and psychological health [78,79,80], the Healthy Parks Healthy People movement, and the Kid’s in the Woods Program, which can harness the power of green spaces to promote human health through outdoor recreation. These initiatives leverage the cultural ecosystem services provided by urban green space to achieve positive outcomes in both the physical and mental health arenas.

Many studies demonstrate a link between green space and local levels of physical activity [44,45,81,82]. In a secondary data analysis of U.S. cities, West *et al.* [44] found that parkland density was positively correlated with physical activity and negatively correlated with obesity. Other reports and literature reviews also suggest that urban green spaces create physical and social environments conducive to healthy, active lifestyles [82,83]. However, some studies reveal mixed relationships between green space and physical activity, with many of these interpretations influenced by cross sectional research designs [84].

The growing fields of environmental psychology and eco-therapy also demonstrate that direct or indirect exposure to natural settings can have a restorative effect on mental health and social interactions [85,86]. Since the stressful qualities of city life can limit leisure opportunities and make city dwellers more susceptible to mental health challenges [87,88], greater exposure to urban green spaces can also alleviate challenges to psychological health. For example, studies show that natural settings in cities can buffer stress or the risk of depression across the United States [89], and on smaller scales including Miami [90], Wisconsin [49], and parts of the Midwest [91]. A study in Michigan found that interactions in nature can positively affect the mood and short term memory of depressed individuals nearly five times as much as non-depressed individuals [50]. Cohen-Cline *et al.* [48] further demonstrated this pattern in Washington. They analyzed the relationship between self-reported depression, anxiety, stress, and access to green space within a 1-km buffer zone of an individual’s home and found a strong inverse relationship between access to green space and depression, yet no statistically significant relationships between green space and stress or anxiety [48]. Many studies have also demonstrated a relationship between exposure to the natural environment and subjective well-being such as happiness [84,92,93,94]. Such findings are important because emotional well-being (e.g., perceived life satisfaction), psychological well-being (e.g., self-acceptance and capacity for personal growth), and social well-being (e.g., sense of community) can be key indicators of mental health, as proposed by the Centers of Disease Control and Prevention [95].

Research also highlights links between physical and mental health that are magnified by nature-based recreation. For instance, outdoor exercise may be an avenue to enhance mood and self-esteem [96]. A review of health outcomes associated with physical activity discovered that outdoor exercise can provide unique contributions to mental health when compared to exercising indoors [83]. Similarly, Wolsko and Lindberg [93] found that outdoor enthusiasts tend to have higher levels of psychological well-being (e.g., subjective vitality, mindfulness, positive emotions, and fewer negative emotions) and a stronger relationship with nature compared to people who are less active outdoors [93]. Even less physically demanding activities in urban green space (e.g., gardening) can promote a positive mood and relieve acute forms of stress [97]. Collectively, these studies illustrate how cultural ecosystem services from urban green space (e.g., nature-based recreation, aesthetic value) can benefit multiple aspects of both physical and psychological well-being.

### 2.2. Neighborhood and Built Environment

Neighborhood and built environment is a social determinant which includes factors such as environmental conditions, access to healthy food, crime, and violence. This is important because a population’s experience of place can have important health implications [2,98]. For instance, Frumkin [99] describes sense of place as the aesthetic, social, physical, spiritual, and psychological qualities of a location that influence one’s attachment and feeling of belonging. Enhancing sense of place can improve one’s sense of self, which encourages positive attitudes and behaviors that are linked to health and well-being [51]. Urban green spaces can foster a sense of place which is also linked to social indicators of health such as community identity and relationship networks [16]. To elaborate, access to green space can improve health by promoting aesthetic surroundings that encourage residents to be more physically active, socialize with neighbors, support mental renewal, and enhance community satisfaction [100].

Evidence suggests that environmental aesthetics may be closely associated with place attachment, or emotional bonds to a location [101]. This link can be facilitated through the perceived quality of green spaces, which can positively relate to sense of community [102] and community attachment among urban residents [52,103]. For example, a cross-sectional study in Denver, Colorado explored the role of neighborhood gardens on neighborhood attachment and discovered that community and home gardens contributed to greater neighborhood attachment [59].

Green spaces may also enhance sense of place and place attachment by increasing neighborhood satisfaction [53]. Florida *et al.* [104] used survey data to examine the influence of aesthetic beauty on community satisfaction across the U.S. and found a significant positive relationship between attractive physical settings (e.g., trails, outdoor parks, and playgrounds) and community satisfaction. A study in College Station, Texas, also noted that characteristics of vegetation (e.g., canopy cover, tree patch size, and shape) can increase neighborhood satisfaction [105]. Along similar lines, other studies have observed a favorable link between coverage of green spaces and social indicators such as neighborhood satisfaction in Flint, Michigan [106], Baltimore, MD [63], and central Ohio [53]. On the contrary, a study in California found that big trees and yard space did not significantly contribute to neighborhood satisfaction [107]. Thus, the influence of nature and vegetation on neighborhood satisfaction can demonstrate mixed results. More research is needed to understand mechanisms driving these relationships.

In addition to sense of place and community satisfaction, cultural services from urban green space can demonstrate more tangible impacts on the neighborhood and built environment. As research highlights the negative health effects of “food deserts” in disadvantaged communities, access to nutritious and affordable foods has become an increasingly important health promotion tool across urban landscapes [108]. The ecosystem services provided by urban green space create opportunities for urban gardening [59] and foraging [60] that contribute to healthy lifestyles. For example, a study in Denver, CO, found that community gardeners consumed significantly more fruits and vegetable daily than their non-gardening counterparts [29]. A similar study in Denver showed that interactions with urban gardens not only increased healthy food intake, but also strengthened residents’ neighborhood attachment and participation in other types of health behaviors [59].

Urban green space also appears to affect rates of crime and other incivilities. For instance, studies in Chicago suggest that greener environments are associated with lower levels of crime [57,109]. Similarly, results from a project in Baltimore suggest that a 10% increase in canopy cover was linked to a 12% decrease in crime [110]. Research in Philadelphia, Pennsylvania, indicates that greening vacant lots can reduce stress as well as decrease gun assaults and acts of vandalism [56]. However, it should be noted that recent literature reviews exploring links between green space and crime reveal mixed results [19,58]. In other words, green space expansion may not always increase favorable health behaviors, particularly when such greening inadvertently threatens public safety [111]. Consequently, the effects of green space on neighborhood and built environments often show spatial variation that is closely tied to the overall social and cultural characteristics of particular locations.

### 2.3. Social and Community Context

The cultural services provided by urban green space can relate to social and community context through factors such as social cohesion and civic participation. While this category overlaps some factors within neighborhood and built environment, our discussion primarily focuses on social interactions among residents that influence health outcomes. Social cohesion pertains to the trust, solidarity, and overall connection among neighbors [59]. Many studies demonstrate how social cohesion can influence a range of factors that are linked to physical and psychological well-being. As an illustration, a study among urban neighborhoods (e.g., Augusta, GA, and Charleston, SC) found that African American women who lived in areas with high social cohesion were less likely to smoke [112]. Social networks can also provide a safety net of support during challenging times [113]. Furthermore, the presence of social support is frequently associated with stress-buffering pathways that are beneficial to cardiovascular, neuroendocrine, and immune function [114]. Because social cohesion is considered a key correlate of health in cities, it is important to identify factors that facilitate social cohesion in urban settings. Many recent studies reveal that accessible neighborhood green space can promote social cohesion and social relationships [52,84,88,115,116]. For example, a project in Australia showed a correlation between perceived greenness and social cohesion in which residents with a higher perception of neighborhood green space had a greater likelihood of improved physical and mental health [62]. Based on these observed associations, the relationships between green space, social cohesion, and health warrant further attention [117].

A review of well-being indices found almost 70 indicators in the literature that provided some means of assessing social cohesion related to ecosystem services [113]. On the other hand, a lack of green space has been linked with feelings of loneliness and low social support [118]. Thus, by encouraging social interactions across diverse populations, green spaces such as public parks can potentially remedy the documented decline of social relationships in urban areas [119]. For example, Fan *et al.* [61] used survey data to examine the role of neighborhood green spaces on social support in Chicago. They found that parks can foster social support and indirectly mitigate stress. In addition, recreation and cultural activities on neighborhood green spaces provide an opportunity for residents to interact with others outside of their family [113]. A study in the UK demonstrated that park visitors who engaged in social activities were more likely to have local acquaintances compared to visitors who used parks for non-social reasons [119]. Larson *et al.*, found that many parents recognized diverse physical, mental, and social health benefits associated with their children’s outdoor recreation experiences in Georgia state parks, particularly when these experiences involved bonding interactions with family and friends [120].

The aforementioned social interactions can positively affect physical and mental health, but they also help promote another important product of cultural ecosystem services: social capital. Social capital refers to networks of support and interaction that facilitate bonding, collaboration, problem solving, and community action [121]. Even though social factors are complex, social capital (*i.e.*, value from social connections) is a viable way to assess neighborhood social relations [63]. For these reasons, social capital is considered a major correlate of community health and well-being and explored in thousands of health-related studies in the past several decades [122]. In cities, these relationships are often mediated by urban green space through either direct or indirect pathways. For example, research shows that exposure to nature and urban green space can lead to enhanced cooperation and sustainable behaviors [64]. Other studies demonstrate strong relationships between time in nature, community attachment, and civic engagement that contribute to health and well-being (e.g., pro-environmental behaviors) [123,124,125]. Along similar lines, studies have found that people who volunteer in green spaces report higher levels of social cohesion and neighborhood social capital than those who do not [126]. Hence, configuring green spaces in ways that enhance social interactions and recreation experiences may be important for maximizing potential health benefits [61].

### 2.4. Education

Education is a social determinant of health which involves indicators such as early childhood education, literacy, high school graduation, and enrollment in higher education [2]. Some studies have linked the benefits from green spaces with aspects of educational achievement and cognitive functioning [39,68,69]. For example, Wu *et al.* [66] examined the relationship between academic performance and surrounding greenness among elementary schools in Massachusetts. After adjusting for confounding variables (e.g., income levels, English not being students’ first language, attendance, gender, and levels of urbanization), the authors determined that higher levels of greenness were associated with higher student performance in English and math [66]. Other research has also observed similar positive links between nearby green space and student performance among high school students in Michigan [67] and school-aged children in Nebraska [65].

Potential explanations for these relationships vary, and may result from nature’s specific impacts on attention restoration, concentration, stress reduction, and social interactions that contribute to youth development [39,127]. For instance, a recent empirical study in Spain found significant relationships between neighborhood greenness and children’s working memory and attentiveness [68]. By positively affecting cognitive capacity [17], contact with nature may ultimately boost educational achievement [68] and promote the long-term health and well-being of urban residents and the communities in which they live.

### 2.5. Economic Stability

Economic stability incorporates indicators of poverty, employment, food security, and housing stability [2]. It influences health by affecting one’s ability to access healthy foods, quality healthcare, and other resources that support the human condition. Many scholars argue that the distribution of urban green space can vary across neighborhoods and provides a reasonable proxy for a community’s socio-economic status [13,128,129]. For example, green space projects can reduce vacant properties that plague many American cities and revitalize communities by creating green jobs, increasing property values, and improving public health [20,56,73]. A study in Philadelphia found that views of local greened lots significantly decreased heart rates when compared to non-green lots, implying that reducing neighborhood blight can minimize stress and enhance human health [20]. Despite these multifaceted benefits—many of which contribute directly to health and well-being—an analysis of greening efforts in seven U.S. cities found that programs were generally framed to address storm water management [130]. From a socio-economic standpoint, the goals of these greening programs could be expanded to integrate cultural ecosystem services and incorporate other dimensions of the urban environment and corresponding health implications. An expanded view of positive externalities would help to ensure that the benefits of urban green spaces are more explicitly integrated into urban economics and policy [19].

Local property values also illustrate the economic impact of urban green spaces. In as study of property values in northern Los Angeles, Conway *et al.* [70] observed that home prices in older urban communities were higher in neighborhoods with greening programs. They also recommend that future studies expand their analysis to include more attributes and values of green space—not just those centered on housing prices [70]. A similar study in New York City compared neighborhood property values within multiple distances of community gardens. They found that gardens have significant positive effects on property values, especially in disadvantaged neighborhoods [71]. Others have also made comparable observations between green spaces and property values in parts of Tennessee [131], Oregon [72], and Wisconsin [132]. Since green spaces can have a positive impact on property values, some imply that investments in green spaces (e.g., gardens) can also benefit property taxes in the surrounding community [71]. These studies suggest that economic stability is closely associated with urban green space, cultivating a relationship that can lead to better health outcomes. Yet questions remain about how these positive outcomes are distributed across diverse populations.

## 3. Environmental Justice and Urban Green Space: A Public Health Perspective

To achieve health equity and promote physical and psychological well-being, it is critical for all communities to have access to the cultural ecosystem services that influence social determinants of health [133]. Despite growing research supporting nature’s role as a health amenity, several review articles show that green spaces and corresponding ecosystem services are not equitably distributed across urban populations [41,134]. A study in Tampa that analyzed the distribution of street trees found that neighborhoods with more low-income households, African Americans, and renters tended to have less tree cover than neighborhoods with more affluent, white, home-owners [76]. Schwarz *et al.* [74] found similar relationships between urban tree cover and median household income in a study across other U.S. cities. However, other studies have questioned reported green-space distribution inequities among certain ethnic or racial groups [135].

Similarly, some research has revealed environmental injustices with respect to urban park distribution [134,136,137], while other studies have not found significant effects of income or race on access to public parks and private recreation facilities [138]. For instance, Wen *et al.* [139] found that although U.S. tracts characterized by poverty and high minority concentrations displayed lower levels of green space coverage, they actually displayed greater spatial access to parks. However, physical proximity and access to parks may not necessarily translate into positive health outcomes, especially if residents are not able to experience the cultural ecosystem services that urban green space provide. In a review of studies examining links between park proximity and physical activity, Bancroft *et al.* [140] found that measures of perceived proximity were more likely to be significantly associated with physical activity than measures of objective proximity. These observations suggest that even if green space is close by, social accessibility (defined by factors such as neighborhood safety and walkability) may represent a substantial barrier to actual use [139]. Inequities also exist with respect to the quality of parks and green spaces. A study by Wright Wendel *et al.* [141] in Santa Cruz, Bolivia, found that while lower-income, peri-urban area residents had access to nearby neighborhood parks, they were not perceived to be as safe and desirable as urban parks closer to wealthier, inner-district residents. Similar disparities were observed in Los Angeles, where disparities in urban park funding have consistently and disproportionately affected green spaces in lower-income neighborhoods [142]. Because lower quality urban green spaces may minimize the extent of amenities and ecosystem services that impact the social determinants of health, populations that use them may enjoy fewer health benefits.

As these trends suggest, environmental justice remains a prominent concern when viewed through the lens of green space amenities and cultural ecosystem services. While the historical environmental justice discourse has centered on disproportionate burdens of environmental health hazards [10,143], the new paradigm focused on nature as a health amenity reveals similar public health concerns. In some cases, efforts to offset these patterns by increasing urban green space gentrifies some communities which displaces residents who can gain the most from these ecosystem services [134]. These concerns extend into the field of public health as ecosystem degradation merges with social disadvantage to create issues that widen gaps in health, especially between socio-economic groups [144]. Approaching public health challenges using ecosystem services can inform strategies to address disparities in physical (e.g., obesity, cardiovascular disease, heat-related illness) and mental (e.g., depression, stress, anxiety) health [14]. For instance, in a recent study in 34 European nations, Mitchell and associates found that access to green space and outdoor recreations areas can reduce gaps in mental well-being among socio-economic groups [145].

Other studies focused on physical health have yielded similar conclusions, demonstrating that public parks associated outdoor recreation opportunities represent a critical physical activity resource in low-income and minority communities [45,46,146]. Since aesthetics, efficacy, and costs of greening projects can influence the extent of local support, engaging low-income residents in green space planning can be beneficial from a policy perspective [147]. Such findings highlight the potential transformative power of urban green space in the public health arena. As social determinants become more prominent in health promotion frameworks, critical links between urban green space and social determinants of health become a fruitful ground for inquiry. Future research on environmental justice can leverage this asset by examining the distributive injustices involving urban nature and cultural ecosystem services.

## 4. Conclusions and Future Research

This article integrates complementary concepts from different disciplines to illustrate how cultural ecosystem services from urban green spaces are linked to equity and social determinants of health. Drawing upon the extensive scholarship that explores the link between nature and health, our synthesis highlights contributions that are widely recognized (e.g., connections between green space and physical activity) and those that warrant further investigation (e.g., links between green space, sense of place, and social capital). The discussion also demonstrates that cultural ecosystem services should not be overlooked or undervalued, for they contribute to the Healthy People 2020 social determinants of health in a variety of ways. Our framework linking urban green space and public health capitalizes on some of the inherent strengths of ecosystems services approach (e.g., interdisciplinary origins, utility as a communication tool) and helps to highlight opportunities for additional progress (e.g., enhanced alignment with existing policies and established methodologies) that were identified in a recent analysis of the ecosystem services framework [148]. Strategically integrating multiple paradigms (*i.e.*, ecosystem services, Healthy People 2020, environmental justice) to address pressing environmental and public health challenges is critical to sustainable development.

Future research should continue to investigate the health impacts of the natural environment (generally) and cultural ecosystem services (specifically). For example, many scholars call for more methods for measuring exposure to nature [84] and the pathways through which nature affects health and well-being [50,149]. Advances on each of these fronts [150] can illuminate more specific mechanisms for enhancing social determinants of population health via urban green spaces. Specifically, future research on urban green space and social determinants of health can explore the following environmental justice themes:

- How are parks and other green spaces distributed *and* utilized across different communities?
- How are cultural ecosystem services perceived and valued among different populations at both the community and household scale?
- What characteristics of urban green space maximize the delivery of cultural services which support social determinants of health?
- To what extent can urban green space and cultural ecosystem services mitigate existing health disparities, and what is the strength of their influence relative to other neighborhood factors (e.g., direct access to health care, poverty, housing conditions)?
- What pathways between humans and interactions with nature lead to positive health outcomes? Do these pathways vary across different populations?
- Does the efficacy of park and/or nature prescription programs vary across socioeconomic groups and/or populations?

Evaluating the role of ecosystem services in the context of environmental justice and urban health and well-being is a complex endeavor, but the task is imperative given society’s challenge with health disparities. Integrative frameworks that bridge disciplinary gaps can inform the science and practice of health promotion, which represents a major opportunity to advance sustainable urban development in innovative ways.
